# The association between OCD and Shame: A systematic review and meta‐analysis

**DOI:** 10.1111/bjc.12392

**Published:** 2022-10-27

**Authors:** Michelle Laving, Francesco Foroni, Madeleine Ferrari, Cynthia Turner, Keong Yap

**Affiliations:** ^1^ School of Behavioural and Health Sciences Faculty of Health Sciences Strathfield New South Wales Australia; ^2^ The Moore Centre South Brisbane Queensland Australia

**Keywords:** meta‐analysis, obsessive–compulsive disorder, OCD, shame, unacceptable thoughts

## Abstract

**Background:**

Due to rumination and self‐criticism over unwanted obsessions and repetitive rituals, shame is a common emotion experienced by individuals with obsessive–compulsive disorder (OCD). Shame is also theorized to have relevance to unacceptable thoughts in OCD. However, empirical research looking at the relationship between OCD and shame is still emerging and findings have been mixed.

**Objectives:**

Our review systematically examines the association of shame with OCD and unacceptable thoughts.

**Methods:**

The last updated search was conducted across five databases between 27 and 29 February 2022. The final selection included 20 papers, 18 of which were used in the primary meta‐analysis to calculate pooled effect sizes between OCD and shame measures using a random effects model. In a separate analysis, three papers were used to calculate pooled effect sizes between shame and OCD symptom dimensions also using a random effects model.

**Results:**

The meta‐analyses identified a significant, moderate and positive correlation between total OCD and shame scores *r* = .352, 95% CI [0.260, 0.438]. In addition, significant, weak and positive relationships were found between shame and three OCD symptom dimensions: unacceptable thoughts *r* = .252, 95% CI [−0.467, 0.9708], harm obsessions *r* = .224, CI [−0.190, 0.638] and symmetry concerns *r* = .200, CI [−0.108, 0.509].

**Limitations:**

Shame measures in the reviewed studies were not specific to OCD, and between‐study variance in the analyses examining unacceptable thoughts was significant.

**Conclusions:**

Our findings support a medium positive relationship between shame and OCD. As shame in OCD can be a barrier to seeking treatment and impair quality of life, it is imperative to address this emotion through psychoeducation, assessment and treatment.

**Practitioner points**

- Individuals with greater levels of identified shame tended to have greater levels of OCD severity.
- The association between shame and four OCD subtypes was significant but weak aside from contamination concerns where a significant relationship was not found.
- Development of a shame scale specific to the experience of OCD will increase our understanding of this relationship and its relevance for clinical work.
- As shame in OCD can be a barrier to seeking treatment and impair quality of life, it is imperative to address this emotion through psychoeducation, assessment and treatment.

## BACKGROUND

Obsessive–compulsive disorder (OCD) is a persistent and incapacitating condition impacting approximately 1% to 3% of the global populace (Kessler et al., 2012). People who suffer from OCD experience a complex interplay of obsessions and compulsions which reinforces the cyclical nature of this condition (Brakoulias et al., 2013). The Diagnostic and Statistical Manual of Psychiatric Disorders‐Fifth Edition (DSM‐5; American Psychiatric Association, 2013) defines obsessions as unwelcome repetitive thoughts, urges, or impulses and compulsions as intentional actions (such as excessive checking, reassurance seeking and avoidance), which are activated to neutralize obsessions and the distress associated with them. Due to the excessive and repetitive nature of these symptoms, they often appear odd and nonsensical to others and can trigger feelings of shame and embarrassment for the OCD sufferer.

A challenge for many with OCD is the occurrence of unacceptable thoughts, a symptom dimension of OCD primarily characterized by taboo or blasphemous themes (e.g., intrusive images or thoughts related to sexual and/or aggressive behaviours or urges; Brakoulias et al., 2013). Also known as autogenous obsessions (Lee & Kwon, 2003), unacceptable thoughts often occur abruptly without an identifiable trigger and are ego‐dystonic in nature. It is estimated that 30% of OCD sufferers experience unacceptable thoughts as their primary OCD symptom (Moulding et al., 2014). Some studies report that OCD patients with unacceptable thoughts experience inferior treatment outcomes (Alonso et al., 2001; Ong et al., 2016) and elevated severity of depression (Yap et al., 2012).

### Shame versus guilt

OCD is associated with several negative emotions that can precede, mediate or develop from the symptoms experienced in this disorder. For example, disgust has been linked to unacceptable thoughts and contamination fears (Olatunji et al., 2015) and feelings of unease and agitation can emerge from ‘Not just right experiences’, a manifestation of OCD involving uncertainty about the satisfactory completion of a task or compulsive ritual (Belloch et al., 2016). More recently, there has been an emergence of research seeking to understand the function of moral emotions like guilt and shame in relation to OCD symptoms, particularly since OCD has been moved from the anxiety disorders classification into the new obsessive–compulsive and related disorders category in the DSM‐5. There is now greater recognition of the role of other emotions in addition to anxiety symptoms (Weingarden et al., 2016).

Shame and guilt are principally viewed as morality‐based constructs, involving a process of self‐reflection and evaluation in relation to social norms (Wolf et al., 2010). Shame is generally conceptualized as an emotion enveloping one's entire being, an experience which feels harder to resolve through restitution or purposeful action (Teroni & Deonna, 2008). Shame can also give rise to feelings of being morally flawed which can precipitate painful feelings and maladaptive coping strategies such as social withdrawal (Wetterneck et al., 2014; Weingarden & Renshaw, 2015).

Shame is typically grouped and measured in two ways: as shame proneness (i.e., trait shame) where individuals tend to be more vulnerable to the consequences of shame (i.e., behavioural transgressions), relative to others without this disposition, and state shame (in the moment shame), which is characterized by occasional and transitory shame reactions in interpersonal contexts (Tangney, 1996; Tangney & Dearing, 2002). Like shame, the dual nature of guilt is highlighted with the inclusion of trait (i.e., maladaptive) and state (i.e., adaptive) guilt subtypes (Cândea & Szentagotai‐Tăta, 2018). Although the events or situations that precipitate feelings of guilt and shame are similar (e.g., perceived moral transgressions), the adaptive function of shame and guilt is highlighted as a key distinguishing factor between these two constructs (Else‐Quest et al., 2012; Tangney & Dearing, 2002). For example, while guilt is commonly characterized by reparative actions in response to a triggering event, the emotion of shame can seem harder to resolve when negative self‐evaluations and judgement from others are internalized (Tangney & Dearing, 2002; Teroni & Deonna, 2008). Nonetheless, it has been argued that fluidity can exist between these constructs, particularly when guilt shifts from its adaptive function and generalizes or overlays with harmful and persistent self‐appraisals (Fergus et al., 2010; Tangney, 1996; Teroni & Deonna, 2008). Despite the conceptual overlap between trait shame (i.e., shame proneness) and trait guilt (i.e., maladaptive guilt), the latter is typically associated with disparate reactions to behavioural transgressions while shame can develop with or without the occurrence of any apparent wrongdoing. An instance relating to shame in OCD would be an individual making negative inferences about their morality and potential to commit violent acts when experiencing unwanted aggressive thoughts.

Research efforts have found guilt to be a key emotion related to OCD symptoms such as contamination concerns, checking behaviours and NJREs (D'Olimpio & Mancini, 2014; Gangemi & Mancini, 2017) and a factor in the persistence of symptoms (Chiang & Purdon, 2019; Mancini & Gangemi, 2015), but the relationship between shame and OCD is less understood. Research attention is required here as several studies have observed greater levels of shame in those with OCD in comparison with healthy controls (Hezel et al., 2012; Kim et al., 2014; Kwak & Lee, 2015; Lochner et al., 2005; Weingarden et al., 2016; Yoosefi et al., 2016). Furthermore, shame has been correlated with decreased quality of life (Singh et al., 2016), elevated levels of suicidality (Ching et al., 2017; Raines et al., 2014), diminished functioning (Weingarden et al., 2016), avoidance behaviours (Visvalingam et al., 2022) and emotion regulation difficulties (Berman et al., 2020; Yap et al., 2017) in OCD samples. It is for these reasons, we focus on shame and exclude measures of trait guilt in our analyses.

Individuals living with OCD Unacceptable Thoughts can experience feelings of shame related to the distressing and ego‐dystonic content of their obsessions which can precipitate concerns with being morally flawed. This can also lead to maladaptive coping strategies such as social withdrawal (Weingarden & Renshaw, 2015), delays in seeking treatment (Glazier et al., 2015) and hesitancy to disclose the nature of symptoms being experienced (Cathey & Wetterneck, 2013; Wheaton et al., 2016). It is therefore important to gain a better grasp of the role that shame plays in OCD, and its implications for research and clinical settings.

### Shame and OCD

There are several theories to account for the occurrence of shame in OCD. Cognitive models of OCD suggest that dysfunctional appraisals around the meaning given to obsessions can lead to a form of cognitive bias known as thought–action fusion (TAF). This model is characterized by two key mechanisms: likelihood and moral TAF (Rachman, 1997). Likelihood TAF involves a level of certitude that the presence of unacceptable thoughts increases the chance that what one fears will transpire (e.g., ‘now that I have had the thought that my wife could become seriously unwell, it will increase the odds of this occurring’). Moral TAF is characterized by a degree of concern that having a taboo thought (e.g., paedophilic obsession) is equivalent to having acted on it.

Recent cognitive behavioural theories of OCD suggest that people can develop feelings of confusion and alarm around their identity when experiencing unacceptable thoughts (Bhar & Kyrios, 2007). For example, the emergence of a ‘feared self’ (i.e., fear of one's character and potential to act on distressing obsessions) can exacerbate the TAF cycle leading to intensified feelings of shame (Valentiner & Smith, 2008) and emotion regulation responses that maintain symptomatic behaviour (Berman et al., 2020).

Cândea and Szentagotai‐Tăta (2018) conducted the first meta‐analysis summarizing the strength of the relationship of shame and guilt with anxiety symptoms, including OCD. Studies published in English language and peer‐reviewed journals with the required data were selected (*n* = 143), and from these, ten reported correlations of shame with OCD. A medium effect size was observed between shame and OCD symptoms (*k* = 10), *r* = .317 (95% CI [0.231, 0.398]) and between partial shame (e.g., pooled variance of shame and guilt) and OCD symptoms (*k* = 4) size, *r* = .272 (95% CI [0.208, 0.334]). While this was the first meta‐analysis to include effect size data on shame with OCD, the reviewers applied a broad search strategy focusing on anxiety symptoms across a range of clinical disorders, excluding grey literature. Therefore, only a small number of studies reporting correlations between shame and OCD were located, two of which used a single item to measure shame. Furthermore, the association between shame and OCD with unacceptable thoughts was not examined. Our systematic review and meta‐analysis will address these gaps with a targeted search strategy focused solely on shame and its association with OCD to offer a more distinct picture of this relationship. Due to the importance of construct validity, only studies reporting correlations from singular shame measures or differentiated shame subscales will be included in our analysis. Findings related to unacceptable thoughts will also be highlighted.

## METHOD

### Eligibility criteria

The review is registered with PROSPERO ID: CRD42019128945. Included studies met the following criteria: (a) employed a quantitative research design, (b) included a psychometrically sound measure of OCD or OCD symptom dimensions, (c) included a psychometrically sound measure of shame or differentiated subscale of shame (trait or state shame measures included). (d) reported a statistical association between shame and OCD at one given time point (i.e., a cross‐sectional design or reported baseline data) or provided this data upon request, (e) recruited either a clinical or non‐clinical sample, (f) participants were aged 5 years or over (paediatric and adult populations) and (g) written in English. These criteria were also applied to the grey literature search.

Regarding point (c), although there is a conceptual overlap between trait guilt (i.e., maladaptive guilt) and trait shame, we have chosen not to include trait guilt subscales in our review as important distinctions remain between the two.

### Data sources and search strategy

The last updated literature search was conducted between 27 and 29 February 2022 in the following electronic databases: *PsycINFO, Web of Science, PubMed, Scopus*, and *Medline Complete* using the following terms: *shame, obsessive compulsive disorder, OCD, Obsessive Compulsive Disorder and Obsessive ‐ Compulsive Disorder* (please see Supporting Information for the detailed search strategy). The ProQuest database was searched for relevant grey literature. Our grey literature search did not extend to conference papers reporting preliminary findings of interest, as the complete report would not be available. A hand search for relevant studies in the included papers was added to this process.

### Study selection

**FIGURE 1:**
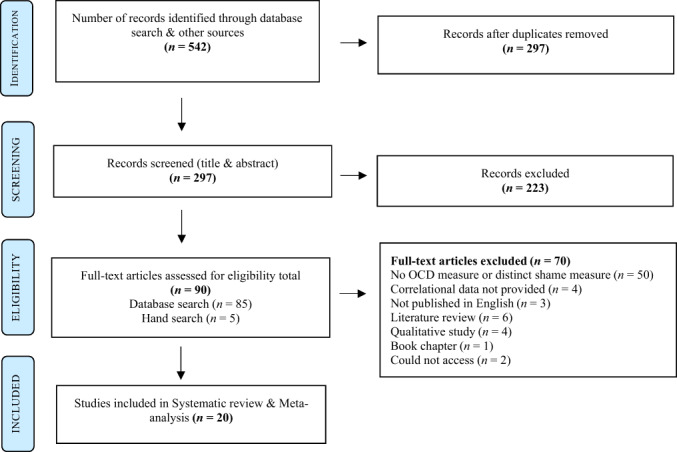
PRISMA flow diagram of selected studies examining the correlation between OCD and shame (Moher, 2009)

Title and abstracts were screened independently by the first author (ML). On occassions where abstracts did not present enough information to determine their suitability, the first author (ML) completed a full‐text review. After completing the read through of studies *n* = 90, the first author (ML) created a table to categorize the papers around the eligibility criteria presented in Figure 1. Of these, *n* = 14 required discussions with a second reviewer (KY) to determine their eligibility for final inclusion. Both reviewers (ML and KY) reached full consensus on the decision to exclude all *n* = 14 studies based on the measures of shame used; therefore, assistance from a third author was not required (Belur et al., 2021). Where studies met all the inclusion criteria but did not report the statistical information required (zero‐order correlations between a shame and OCD measure), we contacted study authors via email to request this information.

### Data extraction

Descriptive data extracted from articles included the primary aim of study, country where sample was obtained, sample size, mean age and standard deviation, sample characteristics, the name of shame and OCD measures used, and zero‐order correlations between a shame and OCD measure. Fifteen authors were contacted to provide correlational data of which nine (60%) responded. Of them, seven provided the data requested while the other two were not able to because they did not use a shame measure in their study because shame measures were not used in their studies. The quantitative data extraction process was conducted independently first by first author* (ML), followed by a review and discussions with the last author (KY) to ensure accuracy.

Risk of bias across for included studies was assessed against the JBI Critical Appraisal Tool for Prevalence Studies (Munn et al., 2015) by two reviewers (ML and KY).

### Data analyses

#### Correlations between shame and OCD

The first analysis pooled effect sizes between total OCD and shame scores across 18 studies. Two studies (Basile et al., 2017; Kwak & Lee, 2015) provided multiple outcomes based on the same participants e.g., zero‐order correlations for three different OCD measures with the Young Schema Questionnaire—Shame/Defectiveness subscale (YSQ; Young et al., 2003). To account for within‐study dependency, a summary effect was calculated for both studies and added to the pooled data to compute an overall effect size (Borenstein et al., 2009; Park & Beretvas, 2019).

The effect size reported is the Pearson Correlation coefficient, *r*, which was converted to Fisher's Z and aggregated using Comprehensive Meta‐Analysis Version 3 software (Borenstein et al., 2013). The random effects model was used for this analysis, which allowed us to observe how the results would generalize to a macrocosm of similar studies (Borenstein, 2019). Heterogeneity (i.e., the amount of observed heterogeneity of actual effects across studies) was reported using the *Q*‐statistic. The prediction interval (i.e., index of dispersion) was calculated to provide a more precise picture of divergence in the true effect size between populations (Borenstein, 2019).

A funnel plot was produced from the primary analysis to get a visual representation of effects from individual study comparisons against a measure of study size. As the primary analysis had more than ten studies, a reasonable variation in sample and effect size and a minimum of one study reporting statistical significance, the requisite criteria were met to apply the trim and fill method (Borenstein, 2019). Using this procedure, we reported an adjusted mean or estimate of what the true versus observed effect would be with bias removed (Duval & Tweedie, 2000).

#### Correlations between shame and OCD symptom dimensions

The second analysis pooled effect sizes between Dimensional Obsessive‐Compulsive Scale (DOCS; Abramowitz et al., 2010) scores for Harm, Symmetry, Unacceptable Thoughts and Contamination with shame measures across three studies.

While several measures of OCD symptom dimensions were included in this review, the DOCS scale was purposely chosen for this analysis as it reflects current research on the characteristics and structure of these dimensions (Abramowitz et al., 2010) and provides a distinct category for Unacceptable Thoughts, thus avoiding convergence with harm obsessions.

One study (Lit, 2017) provided multiple outcomes or non‐independence of effects based on the same participants, for example zero‐order correlations were provided for two shame subscales across each of the four DOCS dimensions. This issue was addressed as it had been in the first analysis, by calculating a summary effect for each of the four DOCS subscales which were then added to the pooled studies to compute a total effect size.

Despite the limited number of studies available for this analysis, we decided to use the random effects model with the Hartung‐Knapp‐Sidik‐Jonkman (Knapp & Hartung, 2003) adjustment to yield a more accurate representation of between‐study variance (Borenstein, 2019; Knapp & Hartung, 2003; Sidik & Jonkman, 2002). The effect size reported is the Pearson correlation coefficient, *r*, which was converted to Fisher's Z and aggregated using Jamovi Version 2.3 software (The Jamovi Project, 2022).

## RESULTS

### Descriptive characteristics of included studies

Table 1 provides a summary of key characteristics from the 20 studies in this review, three of which (Block, 2016; Lit, 2017; Nice, 2013) were unpublished doctoral dissertations at the time of retrieval. Of them, 14 (70%) had a predominantly female sample, four (20%) had mostly male participants, and two (10%) did not provide this information. Most participant samples originated from the United States (55%), and the mean age range across all studies was 18.7 to 40.62 years. Fourteen studies (70%) used a clinical sample, one used a community sample (5%), and five (25%) used undergraduate university students for their study.

**TABLE 1:** Characteristics of included studies

Study and country	Primary study aims	N	Mean age (SD)	% female	Participants	OCD and Shame measures	Correlations between OCD and Shame	r	p
Atalay et al. (2008) Turkey	To examine the onset of early maladaptive schemas in patients with OCD	OCD sample 45	*M* = 31.98 *SD* = 10.58	68.9%	Clinical –psychiatric outpatient	Y‐BOCS, YSQ‐SF (trait shame) Defectiveness/shame EMS	No significant correlation between Y‐BOCS total and YSQ‐SF Defectiveness/shame EMS	.153	.314
							No significant correlation between Y‐BOCS obsession and YSQ‐SF Defectiveness/shame EMS	.114	.455
							No significant correlation between Y‐BOCS compulsion and YSQ‐SF Defectiveness/shame EMS	.16	.293
*Basile et al. (2017) Italy	To explore schemas modes and coping styles in an outpatient OCD sample	OCD sample 34	*M* = 33 *SD* = 8.38	35.29%	Clinical OCD Outpatient clinic	Y‐BOCS, PI‐R, VOCI and YSQ‐ L3 (trait shame) Defectiveness/Shame EMS	No significant correlation between Y‐BOCS total and YSQ‐L3 Defectiveness/shame EMS	−.114	.633
							No Significant correlation between Y‐BOCS Obs and YSQ‐L3 Defectiveness/shame EMS	−.071	.766
							No Significant correlation between Y‐BOCS Comp and YSQ‐L3 Defectiveness/shame EMS	−.433	.057
							No significant correlation between PI total and YSQ‐L3 Defectiveness/shame EMS	.108	>.01
							No significant correlation between VOCI total and YSQ‐L3 Defectiveness/shame EMS	.207	>.01
Block (2016) USA	To explore the relationship between shame, specific irrational and rational beliefs, and cognitive flexibility with OCD and BDD	OCD sample 175	*M* = 24.69 *SD* = 11.75	62.86%	Clinical OCD treatment centres	Y‐BOCS, ESS (trait shame) and OAS (trait shame)	Significant positive correlation between Y‐BOCS total and internal shame ESS	.608	<.01
							Significant positive correlation between Y‐BOCS total and external shame OAS	.623	<.01
							Significant positive correlation between Y‐BOCS total and total shame (ESS and OAS)	.650	<.01
Fergus et al. (2010) USA	To examine the associations between shame and guilt‐proneness with anxiety disorder symptoms	OCD sample 124	*M* = 29.2 *SD* = 13.8	54%	Clinical Intensive outpatient treatment centre	OCI‐R, TOSCA (trait shame)	Significant positive correlation between OCI‐R total and TOSCA Shame ‐proneness	.51	<.05
Fergus and Valentiner (2012) USA	To assess whether scrupulosity reinforces the relationship between concerns about death and four variables of interest (mistake checking behaviour, NJREs, shame and guilt)	92	*M* = 19.7 *SD* = 3.5	59.8%	University students	SCOPI composite SSGS Shame (state shame)	Significant positive correlation between SCOPI‐Core and SSGS Shame	.27	<.01
Haaland et al. (2011) Norway	To explore the relationship between maladaptive schemas and treatment outcome for OCD patients	OCD sample 88	*M* = 34.4 *SD* = 11.5	72.7%	Clinical Psychiatric outpatient	Y‐BOCS, YSQ‐SF (trait shame) Defectiveness/ Shame EMS	No significant correlation between Y‐BOCS total and YSQ‐SF Defectiveness/shame EMS (pre‐treatment)	.07	>.05
*Hezel et al. (2012) USA	To assess pain tolerance in patients with and without moral obsessions and healthy comparison subjects	OCD sample 20	*M* = 20.30 *SD* = 4.86	55%	Clinical group *Residential and intensive outpatient unit at OCD Centre	Y‐BOCS and SSGS (state shame)	Significant positive correlation between Y‐BOCS total and SSGS Shame	.513	<.05
*Kim et al. (2014) South Korea	To evaluate early maladaptive schemas of patients with OCD and clarify relationships between specific EMSs symptom dimensions of OCD and other variables	OCD sample 57	*M* = 26.7 *SD* = 6.3	33%	OCD clinic at a university hospital	Y‐BOCS and symptom checklist and YSQ‐S3 (trait shame) Defectiveness/ shame EMS	Significant positive correlation between Y‐BOCS Obsessions and YSQ‐S3 Defectiveness/shame EMS	.298	<.05
							No significant correlation between Y‐BOCS Compulsions and YSQ‐S3 Defectiveness/shame EMS	.083	.54
							No significant correlation between Y‐BOCS total and YSQ‐S3 Defectiveness/shame EMS	.214	.110
*Kwak and Lee (2015) South Korea	To investigate EMS characteristics among patients with OCD and PAD	OCD sample 51	*M* = 28.0 *SD* = 8.9	31.37%	OCD group Outpatient clinic at a university hospital	Y‐BOCS, MOCI, PI and YSQ‐S3 * Korean version (trait shame) Defectiveness/shame EMS	No significant correlation between Y‐BOCS total and YSQ‐S3 Defectiveness/shame EMS	.092	>.05
							No significant correlation between Y‐BOCS Obsessions and YSQ‐S3 Defectiveness/shame EMS	.211	>.05
							No significant correlation between Y‐BOCS Compulsions and YSQ‐S3 Defectiveness/shame EMS	−.052	>.05
							No significant correlation between MOCI total and YSQ‐S3 Defectiveness/shame EMS	.299	>.05
							No significant correlation between MOCI‐C and YSQ‐S3 Defectiveness/shame EMS	.330	>.05
							No significant correlation between MOCI‐T and YSQ‐S3 Defectiveness/shame EMS	.137	>.05
							No significant correlation between MOCI‐D and YSQ‐S3 Defectiveness/shame EMS	.330	>.05
							No significant correlation between MOCI‐F and YSQ‐S3 Defectiveness/shame EMS	.070	>.05
							No significant correlation between PI total and YSQ‐S3 Defectiveness/shame EMS	.392	>.05
							No significant correlation between PI‐I and YSQ‐S3 Defectiveness/shame EMS	.393	>.05
							No significant correlation between PI‐C and YSQ‐S3 Defectiveness/shame EMS	.291	>.05
							No significant correlation between PI‐U and YSQ‐S3 Defectiveness/shame EMS	.321	>.05
							No significant correlation between PI‐BC and YSQ‐S3 Defectiveness/shame EMS	.067	>.05
Lit (2017) USA	To measure the degree to which guilt and shame, rigid, moralistic thinking and cognitive rigidity contribute to the link between hoarding and scrupulosity	196	*M* = 40.62 *SD* = 11.5	55.6%	MTurk	DOCS, GASP NSE and GASP Withdrawal subscale (trait shame)	*p*‐value not reported for DOCS contamination and GASP NSE correlation	−.08	n/r
							*p*‐value not reported for DOCS contamination and GASP Withdrawal correlation	.19	n/r
							*p*‐value not reported for DOCS Harm and GASP NSE correlation	−.08	n/r
							*p*‐value not reported for DOCS Harm and GASP Withdraw correlation	.22	n/r
							*p*‐value not reported for DOCS Thoughts and GASP NSE correlation	.07	n/r
							*p*‐value not reported for DOCS Thoughts and GASP Withdraw correlation	.27	n/r
							*p‐*value not reported for DOCS Symmetry and GASP NSE correlation	.08	n/r
							*p*‐value not reported for DOCS Symmetry and GASP Withdraw correlation	.16	n/r
*Lochner et al. (2005) South Africa	To conduct a phenomenological comparison between OCD and trichotillomania	OCD sample 55	*M* = 33.1 *SD* = 14.4	46.7%	Clinical (recruited from community primary care practitioners and OCD Association of South Africa)	Y‐BOCS and YSQ (trait shame) Defectiveness/shame EMS	Significant positive correlation between YBOCS total and YSQ Defectiveness/shame EMS	.281	<.05
Malcolm et al. (2021) Australia	To compare identity functioning and internalized shame among BDD, OCD and healthy control participants, and examine clinical correlates in both disorders	OCD sample 22	*M* = 27.0 *SD* = 6.0	63.6%	Clinical	Y‐BOCS and ISS (trait shame)	No significant correlation between YBOCS total and ISS	.39	<.05
Nice (2013) United Kingdom	To examine the relationships between specific self‐concept factors and obsessive–compulsiveness	245	*M* = 30.8 *SD* = 10.41	78.4%	University students	PI‐WSUR and ESS (trait shame)	Significant positive correlation between PI‐WSUR total and ESS	.63	<.01
Olatunji et al. (2015) USA	To determine the degree to which the relationship between shame and manifestations of bulimia and OCD is facilitated by self‐disgust in a large, unselected sample	403	*M* = 19.59 *SD* = 2.47	67%	University students	OCI‐R, OAS (trait shame)	Significant positive correlation between OCI‐R (OCD symptoms) and OAS	.34	<.01
Singh et al. (2016) USA	To explore the relationship between shame, quality of Life and symptom severity in OCRD's	OCD sample 152	n/r	n/r	Clinical (recruited through websites related to OCRDs)	DOCS, ESS (trait shame)	Significant positive correlation between DOCS Total and ESS Character Shame	.19	<.05
							Significant positive correlation between DOCS Total and ESS Behavioural Shame	.16	<.05
							Significant positive correlation between DOCS Total and ESS Total Shame	.20	<.05
							Significant positive correlation between DOCS Symmetry and ESS Behavioural Shame	.16	<.05
							Significant positive correlation between DOCS Symmetry and ESS Total Shame	.16	<.05
Singh et al. (2016) USA (cont'd)							No significant correlation between DOCS Accidental Harm and ESS Character Shame	.11	>.05
							No significant correlation between DOCS Unacceptable Thoughts and ESS Character Shame	.04	>.05
							No significant correlation between DOCS Symmetry and ESS Character Shame	.13	>.05
							No significant correlation between DOCS Contamination and ESS Behavioural Shame	.11	>.05
							No significant correlation between DOCS Accidental Harm and ESS Behavioural Shame	.10	>.05
							No significant correlation between DOCS Unacceptable Thoughts and ESS Behavioural Shame	.01	>.05
							No significant correlation between DOCS contamination and ESS Total Shame	.12	>.05
							No significant correlation between DOCS Accidental Harm and ESS Total Shame	.13	>.05
							No significant correlation between DOCS Unacceptable Thoughts and ESS Total Shame	.03	>.05
*Valentiner and Smith (2008) USA	To investigate why intrusive thoughts are experienced as problematic for some and not for others	700	*M* = 18.7 *SD* = 1.26	61.3%	University students	OCI‐SV, TOSCA (trait shame),	Significant positive correlation between OCI total and Tosca shame scale	.33	<.05
Weingarden and Renshaw (2014) USA	1. Explore whether distorted (OC) beliefs predicted depression severity while controlling for OC symptom severity and 2. To examine shame and guilt as mediators of these associations	263	*M* = 21.06 *SD* = 5.27	77.6%	University students	PI‐WSUR, OBQ‐ 44, TOSCA‐3 SF (trait shame)	Significant positive correlation between PI and TOSCA‐3 SF Shame	.19	<.01
*Weingarden et al. (2016) USA	To examine anxiety and shame as risk factors for depression, suicidality and functional impairment in BDD and OCD	OCD sample 91	*M* = 30.60 *SD* = 10.66	86%	OCD group Clinical	OCI‐R and TOSCA‐4 (trait shame)	Significant positive correlation between OCI‐R total and TOSCA‐4 shame total	.295	<.01
Wetterneck et al. (2014) USA	To investigate the association between shame and OCD dimensions of unacceptable thoughts, harm, contamination and symmetry	OCD sample 90	*M* = 35.64 *SD* = 13.74	74.7%	Clinical *non referred through OCDR‐related websites	DOCS, TOSCA‐3 (trait shame)	Significant positive correlation between DOCS Harm and TOSCA‐3 Shame	.41	<.05
							Significant positive correlation between DOCS Symmetry and TOSCA‐3 Shame	.35	>.05
							No significant correlation between DOCS Contamination and TOSCA‐3 Shame	.10	>.05
							No significant correlation between DOCS Unacceptable Thoughts and TOSCA‐3 Shame	.14	>.05
Yoosefi et al. (2016) Iran	To contrast early maladaptive schemas in patients with OCD and anxiety disorders	Combined OCD, anxiety and healthy controls 151	n/r	n/r	Clinical Psychology and psychiatry clinics	Y‐BOCS, PI‐WSUR YSQ‐SF (trait shame) Defectiveness/shame EMS	Significant positive correlation between PI general score and YSQ‐SF Defectiveness/shame EMS	.54	<.001
							Significant positive correlation between PI‐ COWC and YSQ–SF Defectiveness/shame EMS	.44	<.001
							Significant positive correlation between PI‐ DRGRC and YSQ‐SF Defectiveness/shame EMS	.33	<.001
							Significant positive correlation between PI‐ CHCK and YSQ‐SF Defectiveness/shame EMS	.38	<.001
							Significant positive correlation between PI‐ OTAHSO and YSQ‐SF Defectiveness/shame EMS	.55	<.001
							Significant positive correlation between PI‐ OITHSO and YSQ‐SF Defectiveness/shame EMS	.49	<.001

*Note*: **OCD measures:** DOCS = Dimensional Obsessive‐Compulsive Scale (DOCS Symmetry, DOCS Contamination, DOCS Harm, DOCS Unacceptable Thoughts); MOCI = The Maudsley Obsessional‐Compulsive Inventory (MOCI‐C = checking, MOCI‐T = tidiness, MOCI‐D = doubting, MOCI‐F = fear of contamination); OCI‐SV = The Obsessive‐Compulsive Inventory‐Short Version; OCI‐R = The Obsessive‐Compulsive Inventory‐Revised; PI = The Padua Inventory (PI‐C = checking behaviours, PI‐I = impaired control over mental activities/doubting, PI‐BC = contamination, PI‐U = worries about losing control over motor behaviour); PI‐R = The Padua Inventory‐Revised Version; PI‐WSUR = Padua Inventory Washington State University Revision (COWC = Contamination obsessions and washing compulsions, DRGRC = dressing/grooming compulsion, CHCK = Checking compulsions, OTAHSO = Obsessional thoughts about harm to self/others, OITHSO = Obsessional impulses to harm self/others); SCOPI composite = Schedule of Compulsions, Obsessions and Pathological Impulses; VOCI = Vancouver Obsessive‐Compulsive Inventory; Y‐BOCS = Yale‐Brown Obsessive‐Compulsive Scale; Y‐BOCS checklist = Yale‐Brown Obsessive‐Compulsive Symptom Checklist.

**Shame measures:** ESS = Experience of Shame Scale (ESS Behaviour; ESS Character); GASP = The Guilt and Shame Proneness Scale (GASP NSE = Negative Self‐Evaluation, GASP Withdrawal); ISS = Internalized Shame Scale; OAS = Other as Shamer; TOSCA = The Test of Self‐Conscious Affect; TOSCA‐3 SF = The Test of Self‐Conscious Affect—Version 3 Short Form; TOSCA‐4 = The Test of Self‐Conscious Affect—Version 4; SGSS = State Shame and Guilt Scale; YSQ‐L3 = Young Schema Questionnaire—Long Version, YSQ‐SF = Young Schema Questionnaire Short Form (Shame/Defectiveness EMS = Shame/Defectiveness Early Maladaptive Schema); YSQ‐S3 = Young Schema Questionnaire Short Form—Version 3 (Shame/Defectiveness EMS = Shame/Defectiveness Early Maladaptive Schema); YSQ‐S3 = Young Schema Questionnaire Short Form—Version 3 *Korean version (Shame/Defectiveness EMS = Shame/Defectiveness Early Maladaptive Schema).

n/r = not reported.

Studies marked with an asterisk provided correlational data upon request.

Confirmation of an OCD diagnosis was sought by seven studies, six of which used versions of the Structured Clinical Interview for Diagnosis of DSM IV Mental Disorders (SCID‐1; First et al., 1997; SCID‐1/P V2; First et al., 1998; SCID‐1/P; First et al., 2002a; SCID‐I/NP; First et al., 2002b) and one study used The Mini‐International Neuropsychiatric Interview (M.I.N.I.; Sheehan et al., 1998).OCD symptom severity was measured by 15 studies with the majority (*n* = 10) using the Yale‐Brown Obsessive‐Compulsive Scale (Y‐BOCS; Goodman, 1989).

Five studies (25%) provided correlations between shame and OCD symptom dimensions, with the majority (*n* = 3) using the DOCS (Abramowitz et al., 2010).

The most frequently used measure of shame was the Young Schema Questionnaire * Shame/Defectiveness subscale (YSQ; Young et al., 2003) used by seven studies (35%) followed by various versions of the Test of Self‐Conscious Affect (TOSCA; Tangney et al., 1989) shame subscale which was used by five studies. Eighteen studies measured trait shame (i.e., shame proneness), while only two (Fergus & Valentiner, 2012; Hezel et al., 2012) measured state shame (i.e., in the moment shame) using the State Shame and Guilt Scale (SSGS; Marschall et al., 1994). Thirteen studies used statement‐based measures of shame, while the other seven used scenario‐based measures.

### Meta‐analysis: Shame and OCD

**FIGURE 2:**
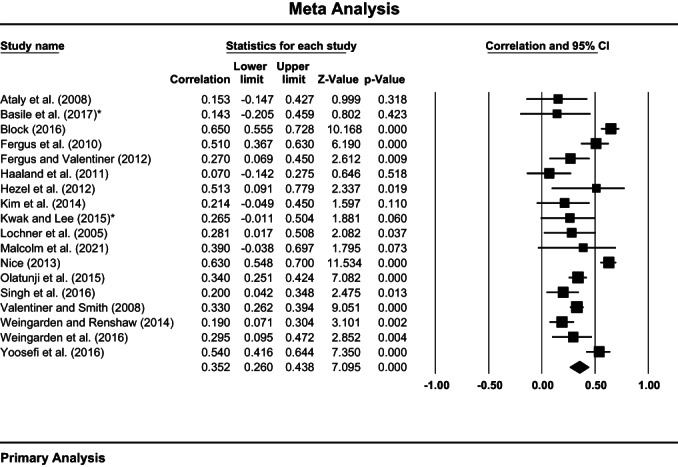
A summary effect was calculated for studies marked with an asterisk

Results of the meta‐analysis are presented in Figure 2. A significant and medium positive correlation was found between OCD symptoms and shame across eighteen studies *r* = .352, 95% [CI: 0.260, 0.438]. A variance of 83.22% from the observed effect size is reflected in the true effect size (*I* ^2^ = 83%, *Q*‐value = 101.32, *T* ^2^ = 0.035, *T* = 0.188). The variation of effect size across studies indicated through the 95% prediction interval is 0.260 (lower limit) to 0.438 (upper limit).

### Shame with OCD DOCS Subscales

A significant but weak positive correlation was found between shame and *OCD unacceptable thoughts r* = .252, 95% CI [−0.4671, 0.9708] with 91% of the variance from the observed effect size being reflected in the true effect size (*I* ^2^ = 90.97%; *Q*‐value = 27.361, *T* ^2^ = 0.0739, *T* = 0.272). A significant but weak positive correlation was found between shame and *OCD harm* scores *r* = .224, 95% CI [−0.190, 0.638], with 73% of the variance from the observed effect size being reflected in the true effect size (*I* ^2^ = 73.29%; *Q*‐value = 6.192, *T* ^2^ = 0.0201, *T* = 0.142). A significant but weak positive correlation was found between shame and *OCD symmetry* scores *r* = .200, CI [−0.108, 0.509], with 56% of the variance from the observed effect size reflected in the true effect size (*I* ^2^ = 56.38%; *Q*‐value = 3.717, *T* ^2^ = 0.0095, *T* = 0.097). A correlation of significance was not found between shame and *OCD contamination* scores *r* = .0875, CI [−0.002, 0.177], with 2% of the variance from the observed effect size reflected in the true effect size (*I* ^2^ = 1.6%, *Q*‐value = 0.368, *T* ^2^ = 0.0001, *T* = 0.011). Although we observed a positive effect size for unacceptable thoughts, harm and symmetry symptom dimensions, after applying the Knapp Hartung Adjustment, results indicate that the true effect size may not be significant in comparable studies.

### Publication bias

**FIGURE 3:**
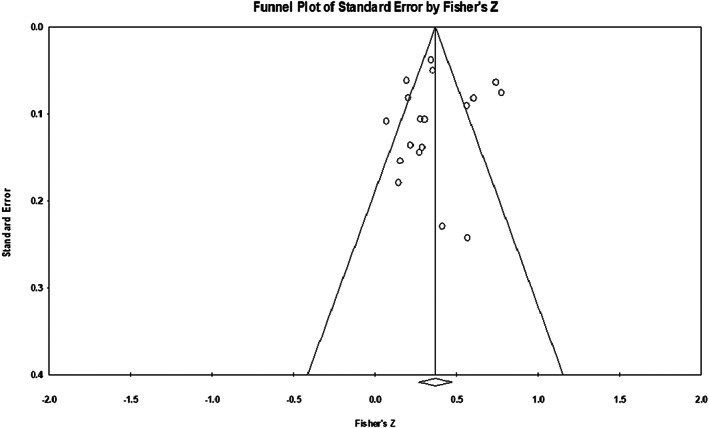
OCD with shame total scores—funnel plot of observed studies

A representation of observed studies (shame in OCD) in the funnel plot below revealed the presence of asymmetry with most studies falling on the left side of the vertical axis (see Figure 3). The trim and fill analysis (Duval & Tweedie, 2000) was used to look for omitted studies or those that could be missing due to publication bias. Employing the random effects model, the point estimate after values had been adjusted is *r* = .35231, 95% [CI: 0.26027, 0.43801]. Although the Trim and Fill output assumes that the pattern of effects is related to publication bias, it is worth noting that it may also be influenced by other factors such as the validity of measures used, the appropriate use of statistical analysis and sampling factors (Borenstein, 2019).

### Risk of bias across studies

Risk of bias was assessed by two reviewers (ML and KY) to provide additional context to the primary analyses. Items one, two, four, seven and eight from the JBI Critical Appraisal Tool for Prevalence Studies (Munn et al., 2015) were used for this assessment. Each question was rated with Yes, No, Unclear or Not applicable with final consensus being reached through discussion. In studies where correlations were requested, (*n* = 7) information pertaining to how their data were collected and statistically analysed was not available. Consequently, they were excluded from this appraisal leaving (*n* = 13) studies reviewed for risk of bias.

Item one (criteria for inclusion in the sample clearly defined) was not applicable for seven studies that used unselected or self‐identified samples and the remaining (*n* = 6) met the criteria. Item two (study subjects and the setting described in detail) was not applicable for one study that used a self‐identified sample, and the remaining (*n* = 12), described the study participants and setting in detail. Criteria were met for item four (objective, standard criteria used for measurement of the condition), item seven (outcomes measured in a valid and reliable way) and item eight ( appropriate statistical analysis used) in all (*n* = 13) studies.

## DISCUSSION

Cognitive models of OCD propose that feelings of shame may emerge from obsessional themes that perpetuate negative self‐beliefs, however studies reporting correlations between shame and OCD have provided mixed results. This study sought to review and synthesize combined effect sizes from studies providing correlational data on the observed relationship between shame, OCD and unacceptable thoughts. Overall, we found a medium and significant correlation between shame and OCD symptom severity (*r* = .352), consistent with current research (Cândea & Szentagotai‐Tăta, 2018). This indicates that there is a significant likelihood that individuals who experience OCD symptoms will also experience shame.

This study also investigated the aggregate correlation of shame with four primary OCD symptom dimensions (Harm, Unacceptable Thoughts, Symmetry and Contamination). Weak and significant correlations were observed for three dimensions; *Unacceptable Thoughts* (*r* = .252), OCD *Harm* scores (*r* = .224) and OCD Symmetry (*r* = .200); however, no relationship was found between shame and *OCD Contamination* scores (*r* = .0875).

The significant albeit weak relationship between shame and DOCS *Unacceptable Thoughts* may have been influenced by the shame measures used in this review as they were not conceptualized to tap into the specific expression of shame in this symptom dimension. Moreover, as disclosure of unacceptable thoughts can be met with higher levels of social rejection compared with other symptoms such as contamination concerns (Cathey & Wetterneck, 2013), it is worth considering the potential impact of social desirability bias in these results. A similar correlation was observed for the DOCS Harm dimension. Given that obsessions with harm themes are also experienced in unacceptable thoughts, it is possible that factors influencing this OCD dimension were also relevant for harm symptoms in our analysis. For example, in one study, responses from a community sample to OCD presentations of harm and symmetry concerns reported higher levels of stigmatizing attitudes for harm obsessions when the participants' OCD diagnosis was not disclosed (Homonoff & Sciutto, 2019). Harm obsessions have also been rated as more unacceptable than OCD washing and checking behaviour by an undergraduate sample (Simonds & Thorpe, 2003).

The positive but weak relationship between shame and OCD Symmetry may be understood in the context of perfectionistic striving which can be activated to manage discomfort with ‘Not Just Right Experiences’ and feelings of incompleteness that are associated with symmetry concerns (Pinto et al., 2017). As shame is theoretically linked to perfectionism (Wetterneck et al., 2014), these factors may provide an explanation for the association we observed.

A non‐significant relationship was observed for shame with the DOCS *contamination* subscale, which contains four questions related to thoughts and experiences around contamination concerns. A possible explanation for this result could be that contamination concerns are typically attributed to an external cause, whereas internal triggers are more often connected to feelings of shame (Lee & Kwon, 2003) or it may be that guilt is the emotion more commonly experienced here as reparative actions can be made to mitigate these concerns (Cougle et al., 2011; Moulding et al., 2014). Contamination symptoms also tend to be more widely associated with OCD in the general population and therefore may be experienced as less shameful for sufferers (Cathey & Wetterneck, 2013); however, contrary findings have been reported in another study (Steinberg & Wetterneck, 2017).

### Limitations

There are limitations in this review worth noting. First, the research focus of the studies was diverse, with few having OCD and its relationship with shame as the primary emphasis (e.g., Fergus et al., 2010; Wetterneck et al., 2014). Therefore, the measures or subscales used were not representative of the shame experience in OCD. This limitation was discussed by Wetterneck et al. (2014) who investigated the association of dispositional shame (i.e., shame proneness) in symptom domains of OCD using the Test of Self‐Conscious Affect‐3 (TOSCA‐3; Tangney et al., 2000). This shame measure asks participants to respond to hypothetical situations related to external events. The nature of shame assessed by the TOSCA‐3 might be inconsistent with OCD‐specific shame, which is often triggered by the internal experience of obsessions. The authors suggested that this methodological flaw may be a reason why a significant relationship between unacceptable thoughts and shame proneness was not observed in their study. There is clearly a need for additional primary research into the experience of shame for OCD sufferers.

Most studies included in this review used self‐report instruments to assess shame, and while this may reduce the risk of under or over‐reporting that can occur in clinician‐administered interviews, there are some disadvantages worth noting (Rüsch et al., 2007). First, variability in the way individuals interpret the wording of a questionnaire and the choices involved in rating their responses can be an issue. Furthermore, given the often crippling and private experience of aggressive/sexual obsessions and feelings of shame, social desirability response bias may occur and influence the internal and external validity of study data (Clerkin et al., 2014).

The use of tacit measures, such as the Implicit Association Test (IAT; Greenwald et al., 1998) where unconscious and instinctive responses, attitudes and beliefs are recorded, could be a feasible measurement of OCD‐related shame. For example, nonverbal coding has the potential to capture automatic shame responses and mitigate the limitations arising from the use of self‐report measures alone (see Clerkin et al., 2014). However, like the shame measures included in this review, IAT stimuli may not be specific to the shame triggers commonly experienced by OCD sufferers and implicit association tests are limited to assessing state shame and not traits. (Clerkin et al., 2014; Robins et al., 2007).

Although the random effects model was applied to adjust for between‐study variations in the primary analysis, the potential role of other variables and patterns of heterogeneity have not been accounted for, therefore, caution should be used when making conclusions about the true effect size between shame and OCD in any single population. For example, while it is tempting to conclude that the experience of OCD obsessions alone induces feelings of shame, it is possible that individuals may experience shame for unrelated reasons or due to comorbid conditions such as depression which has been positively correlated to shame in a meta‐analytic review (Kim et al., 2011) and can develop as a secondary condition after OCD symptoms have emerged (Weingarden et al., 2016; Yap et al., 2012). As co‐occurring conditions such as depression were not reported in all studies, we did not conduct a moderator analysis to account for its potential contribution to the results (Wetterneck et al., 2014). Furthermore, we cannot make inferences about the causal direction of shame and OCD from our analysis and potential mediating factors such as self‐disgust (Olatunji et al., 2015). Expanding research efforts using both experimental and longitudinal designs to investigate the direction of causality would enrich our understanding of this relationship.

In the second analysis, there were limitations in the statistical models available to us because of the small number of studies we were working with. Using the random effects model with the KHSJ adjustment provided a more accurate confidence interval; however, the extent of the adjustment was substantial across all symptom dimensions, as such, we cannot provide a reliable or meaningful estimation of the mean effect size for comparable studies (Borenstein, 2019). The development of an OCD‐specific shame scale, followed by research examining the association of shame with all four DOCS symptom dimensions, using a significantly larger number of studies, could address this limitation.

Experimental studies using OCD obsession induction scenarios, followed by self‐report measures of shame could be another option. An experimental study conducted by Visvalingam et al. (2022) used inductions for harm, sexual, contamination and symmetry obsessions followed by a self‐report rating, finding sexual obsessions and harm produced the highest levels of shame. Although participants were tentative in their participation with the induction of taboo thoughts, the additional reporting on compulsion compulsions plural and avoidance behaviours was a novel way of increasing the study's ecological validity (i.e., behaviour in the study is more predictive of behaviours *in situ*).

While OCD has a biological predilection, environmental influences such as culture may also influence obsessional themes, the expression of symptoms, and associated emotions such as shame (Yakeley, 2018). Consequently, exploring the role of cultural dynamics in this relationship would be a key consideration in developing an OCD‐specific shame measure.

### Implications for future research

Considering the theoretical support for symptom‐based shame in unacceptable thoughts (Weingarden & Renshaw, 2014), it is surprising that empirical research in this area is lacking. The development of a valid measure to improve our understanding of how shame is uniquely experienced in the context of behaviour, cognitions and self‐appraisal within this symptom dimension, could address the key methodological limitations in this review. Qualitative studies gathering phenomenological data to inform the development of a validated shame measure is a suggested approach.

Finding that shame was associated with all symptom subtypes aside from contamination concerns raises some important clinical considerations. Firstly, it seems important to provide more widespread information about the experience of shame, especially as it can be one of the factors influencing whether people seek treatment. There are increasing numbers of podcasts, websites, and webinars available, seeking to give information about OCD, and a psychoeducational focus on the role of shame in OCD and in help‐seeking behaviour could be of benefit (Marques et al., 2010; Singh et al., 2016).

In standard treatments like cognitive behavioural therapy (CBT) and exposure with response prevention (E/RP), clients are supported to disclose obsessional themes and content as part of their treatment. If shame is a barrier to sharing this information, important cognitive restructuring and E/RP targets cannot be achieved. To this point, establishing therapeutic rapport first and normalising the presence of Unacceptable Thoughts in OCD may help facilitate the disclosure of unacceptable thoughts, especially when they are associated with elevated levels of shame Weingarden & Renshaw (2015).

The inclusion of integrative models such as schema therapy (Young et al., 2003) may also be beneficial in the treatment of OCD considering several studies have reported higher levels of the shame/defectiveness schema in OCD compared with control groups (Atalay et al., 2008; Kim et al., 2014; Kwak & Lee, 2015). Schema therapy was originally developed to help clients with trauma and personality disorders recognize and address maladaptive schemas formed through harmful early life experiences. It has been suggested that schema therapy might be an effective treatment option to gain insight and relief from shame‐inducing OCD obsessions (Kizilagac, 2019) that originate from or are compounded by early developmental experiences (Haaland et al., 2011).

Schema therapy augmented with E/RP has shown preliminary effectiveness in reducing OCD symptom severity with a small sample of patients who have not benefited from CBT previously (Thiel et al., 2016); however, as far as we know, research examining its effectiveness in addressing shame across the scope of OCD symptom dimensions has not been conducted. As unacceptable thoughts are characterized by morality‐based concerns and negative self‐evaluations, it would be constructive to design research that explores the potential role the shame/defectiveness schema plays in the progression of these symptoms (Peeters et al., 2021). Another therapeutic approach that could offer clinical value in managing shame in OCD is compassion‐focused therapy (CFT; Gilbert, 2014). CFT was developed to assist individuals experiencing heightened shame and self‐criticism across a range of psychological contexts. This treatment approach has demonstrated efficacy in reducing shame through developing the self‐regulatory system and the practice of self‐compassion (Ferrari et al., 2019; Kirby et al., 2017). CFT also highlights the influence of threat‐related drives within the self‐regulatory system which can be formed in early development, not dissimilar to maladaptive schemas.

A recent pilot study conducted by Petrocchi et al. (2021) evaluated the tolerability and effectiveness of an 8‐week CFT program to decrease OCD symptoms and accompanying concerns such as fear of guilt and self‐criticism with a small sample (*n* = 8) of OCD patients who had completed a minimum of 6 months CBT treatment prior with little improvement. Participants reported a decrease in OCD symptoms, fear of guilt and self‐criticism and satisfaction with the treatment. However, these outcomes are not applicable to our theoretical understanding of shame in OCD and additional research is required to examine the components of CFT that would be most effective here. Examining the utility of developing emotional regulation around OCD‐related shame in the therapeutic setting could be of value, particularly in cases where suppression of intrusive and unsettling thoughts is a primary concern (Chase et al., 2019; Yap et al., 2017). As an example, using regulation techniques prior to E/RP interventions may lower the distress related to shame‐inducing obsessions and potentially increase willingness to engage in certain exposure targets. Likewise, work on self‐compassion alongside emotion regulation techniques could cultivate more acceptance of uncomfortable feelings such as shame, during various treatment phases.

Finally, it would be beneficial if CFT could tap into the dimension‐specific characteristics of OCD to optimize therapeutic outcomes. For example, with Unacceptable Thoughts some individuals feel compelled to maintain compulsions as an act of contrition when experiencing obsessions with repugnant themes (Moulding et al., 2014). Research exploring the role of shame with self‐punishment behaviours (Fergus et al., 2010; Jacoby et al., 2015) could inform therapists of potential barriers to the successful delivery of CFT.

## CONCLUSION

This systematic review and meta‐analysis focus on the strength of the association between shame, OCD and unacceptable thoughts. Our findings showed a medium aggregate positive correlation between OCD severity and shame, and a similar albeit weaker association between shame and three common OCD symptom subtypes (*Unacceptable Thoughts, Harm and Symmetry*). Future research, addressing the noted methodological and conceptual limitations in this review, will prove useful in expanding our understanding of this relationship and its relevance for clinical work.

## AUTHOR CONTRIBUTIONS

**Michelle Laving:** Conceptualization; formal analysis; investigation; methodology; validation; visualization; writing – original draft; writing – review and editing. **Francesco Foroni:** Supervision; writing – review and editing. **Madeleine Ferrari:** Formal analysis; writing – review and editing. **Cynthia Turner:** Supervision; writing – review and editing. **Keong Yap:** Conceptualization; formal analysis; methodology; supervision; validation; writing – review and editing.

## CONFLICT OF INTEREST

All authors declare no conflict of interest.

## Supporting information


Supporting Information

## Data Availability

Data sharing is not applicable to this article as no data sets were generated or analysed during the current study.
