# Supplementary material for: The association between OCD and Shame: A systematic review and meta‐analysis
Source: Br J Clin Psychol. 2022 Oct 27;62(1):28–52. doi: 10.1111/bjc.12392 (PMC10091722; doi:10.1111/bjc.12392)
Supplement: Supplementary file 1 — Supporting Information [file BJC-62-28-s001.pdf]

MEDLINE	Ebsco Host	(DE "Obsessive Compulsive Disorder" OR OCD OR "obsessive?compulsive disorder*") AND ((DE "Shame") OR (Shame*)
Psych INFO	Ebsco Host	(DE "Obsessive Compulsive Disorder" OR OCD OR "obsessive?compulsive disorder*") AND ((DE "Shame") OR Shame*)
Scopus	Elsevier	((TITLE-ABS-KEY (shame)) OR (TITLE-ABS-KEY (" shame"))) AND ((TITLE-ABS-KEY (ocd OR "Obsessive Compulsive Disorder*")) OR (TITLE-ABS-KEY ({Obsessive - Compulsive Disorder}))
Web of Science	Clarivate Analytics	(TS = (shame*) OR (TS = (“shame”) AND (OCD OR “Obsessive? Compulsive Disorder*) OR (TS = “obsessive Compulsive Disorder”)
PubMed	NCBI	("obsessive compulsive disorder"[MeSH Terms] OR (OCD[All Fields] OR "Obsessive? Compulsive Disorder*"[MeSH Terms])) AND (shame[All Fields] OR shame'[All Fields]
